# Study of the Relationships between the Structure, Lipophilicity and Biological Activity of Some Thiazolyl-carbonyl-thiosemicarbazides and Thiazolyl-azoles

**DOI:** 10.3390/molecules201219841

**Published:** 2015-12-11

**Authors:** Radu Tamaian, Augustin Moţ, Radu Silaghi-Dumitrescu, Ioana Ionuţ, Anca Stana, Ovidiu Oniga, Cristina Nastasă, Daniela Benedec, Brînduşa Tiperciuc

**Affiliations:** 1 National Research and Development Institute for Cryogenic and Isotopic Technologies, 4th Uzinei Street, Râmnicu Vâlcea 240050, Romania; radu.tamaian@icsi.ro; 2 3Nano-SAE Research Centre, Faculty of Physics, University of Bucharest, P. O. Box MG-38, Bucharest-Măgurele RO-077125, Romania; 3 SC Biotech Corp SRL, 4th Uzinei Street, Office C52, 2 Râmnicu Vâlcea 40050, Romania; 4 Department of Chemistry and Chemical Engineering, Babeş-Bolyai University, 1st Mihail Kogălniceanu Street, Cluj-Napoca RO-400084, Romania; rsilaghi@chem.ubbcluj.ro; 5 Department of Pharmaceutical Chemistry, Faculty of Pharmacy, Iuliu Haţieganu University of Medicine and Pharmacy, 41 Victor Babeş Street, Cluj-Napoca RO-400012, Romania; ionut.ioana@umfcluj.ro (I.I.); teodora_anca@yahoo.com (A.S.); onigao65@yahoo.com (O.O.); cmoldovan@umfcluj.ro (C.N.); brandu32@yahoo.com (B.T.); 6 Department of Pharmacognosy, Faculty of Pharmacy, Iuliu Haţieganu University of Medicine and Pharmacy, 12 Ion Creangă Street, Cluj-Napoca RO-400010, Romania; dani_67ro@yahoo.com

**Keywords:** thiosemicarbazide, thiazolyl-azole, lipophilicity, PCA, anti-inflammatory, antioxidant

## Abstract

Lipophilicity, as one of the most important physicochemical parameters of bioactive molecules, was investigated for twenty-two thiazolyl-carbonyl-thiosemicarbazides and thiazolyl-azoles. The determination was carried out by reversed-phase thin-layer chromatography, using a binary isopropanol-water mobile phase. Chromatographically obtained lipophilicity parameters were correlated with calculated log P and log D and with some biological parameters, determined in order to evaluate the anti-inflammatory and antioxidant potential of the investigated compounds, by using principal component analysis (PCA). The PCA grouped the compounds based on the nature of their substituents (X, R and Y), indicating that their nature, electronic effects and molar volumes influence the lipophilicity parameters and their anti-inflammatory and antioxidant effects. Also, the results of the PCA analysis applied on all the experimental and computed parameters show that the best anti-inflammatory and antioxidant compounds were correlated with medium values of the lipophilicity parameters. On the other hand, the knowledge of the grouping patterns of the tested variables allows the reduction of the number of parameters, determined in order to establish the biological activity.

## 1. Introduction

Lipophilicity is an important physicochemical property of bioactive compounds affecting their biological activity with a determinant role in the transport of compounds through biological membranes and in the formation of the ligand-receptor complex. The lipophilic character of an active molecule, defined as the ability of a compound to penetrate through hydrophobic barriers in order to get from the delivery point to the site of action [1,2], is usually quantitatively characterized as the logarithmic forms of the *n*-octanol-water partition coefficient P_ow_ (LogP_ow_) and *n*-octanol-water distribution coefficient D_ow_ (LogD_ow_)—experimentally determined [3] or calculated by using a series of mathematical models [4]. Due to experimental limitations of the classical “shake-flask” method [5], the most widely used techniques for the measurement of the lipophilic properties of different chemical molecules are nowadays the chromatographic techniques in reversed-phase systems (RP-TLC and RP-HPLC) [6,7].

Some of the parameters resulting from a RP-TLC may be associated to the lipophilic character of the analytes [8,9]. The retention parameters (R_M_) are calculated by means of well-known Batte-Smith and Westall Equation (1):

R_M_ = log (1/R_F_ − 1) (1)

Generally, the R_M_ values determined by RP-TLC are linearly dependent on the concentration of the organic modifier (C) in the mobile phase Equation (2):

R_M_ = R_M0_ + bC (2)

where b and R_M0_ are, respectively, the slope and the intercept of Equation (2). Extrapolation of the R_M_ value to pure water, leads to determination of R_M0_, which can be considered as an estimation of the partitioning of compounds between nonpolar stationary phase and the aqueous system, and hence a way to estimate the lipophilicity of the compounds (Soczewinski-Wachtmeister model) [9,10]. Additionally, it has been stated that not only the R_M0_, but also the slope, b, as a characteristic of the specific hydrophobic surface area of the compounds, can be used as an estimation of lipophilicity [6,11,12]. The advantages of TLC methods consist in the small quantity of compounds needed for the determination, and the rapidity and simplicity of the method. Also, they are inexpensive, rapid and easy to perform [13].

Principal Component Analysis (PCA) is a chemometric tool designed to transform a set of original variables into new uncorrelated variables (axes), which are called principal components. The new variables are linear combinations of the original variables and the new axes lie along the directions of maximum variance. PCA provides an objective way of finding indices of this type, so that the variation in the data can be accounted for, as concisely as possible. By applying PCA to the matrix formed by the retardation factors (R_F_) of all compounds in all mobile phases, it is possible to obtain a new lipophilicity scale where the linear combinations of retention indices (the scores) corresponding to the first component (PC1) appears to be a new parameter capable to quantitatively assess the lipophilic character of the compounds [6,14,15,16].

Based on our experience in the field of heterocyclic compounds [17,18,19], we synthesized and evaluated *in vivo*, in term of anti-inflammatory and antioxidant activity, a new series of thiazolyl-carbonyl-thiosemicarbazides and hybrid thiazolyl-1,3,4-oxadiazoles, thiazolyl-1,3,4-triazoles and thiazolyl-1,3,4-triazoles [20,21]. The results demonstrated that the new thiazole compounds exhibit anti-inflammatory effects, lowering the acute-phase response of bone marrow and the oxidative stress. Based on the virtual screening and on the obtained results, the activity may be due to their capacity to reduce NO synthesis by blocking the binding of l-arginine at the active site of inducible nitric oxide synthase (iNOS) [21].

The goal of the present study was to determine the relationships between the computed lipophilicity coefficients and RP-TLC retention parameters of new thiazolyl-carbonyl-thiosemicarbazides and thiazolyl-azoles with the anti-inflammatory/antioxidant potential and their structural parameters obtained by molecular modeling calculations, applying the PCA method.

## 2. Results and Discussion

### 2.1. Chemistry

**Figure 1:**
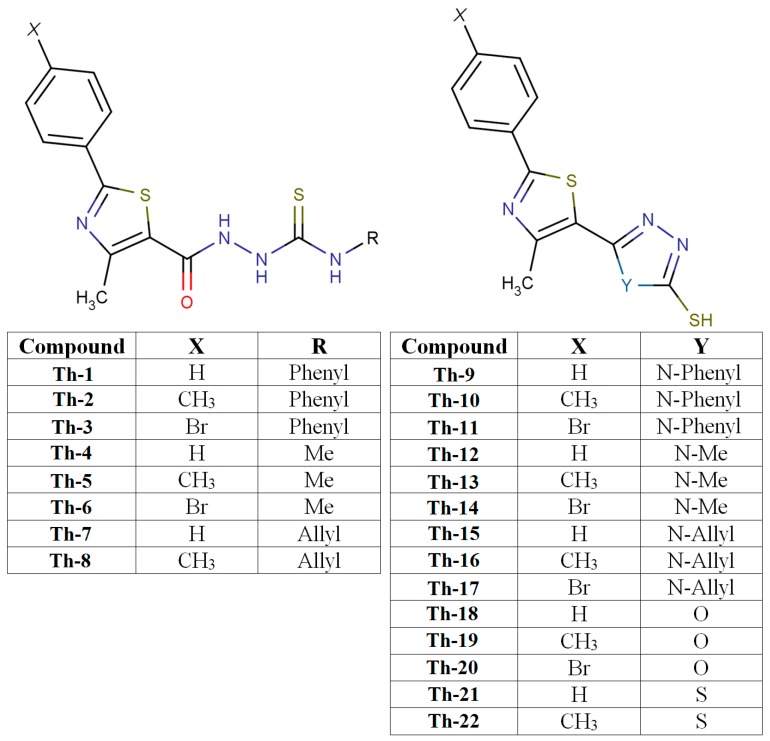
Structures of the studied thiazolyl-carbonyl-thisemicarbazides and thiazolyl-azole compounds—Marvin was used for drawing and displaying of chemical structures (MarvinSketch 6.3.1, 2014, ChemAxon Kft., Budapest, Hungary, http://www.chemaxon.com).

Twenty two thiazolyl-carbonyl-thiosemicarbazides and hybrid thiazolyl-1,3,4-oxadiazoles, thiazolyl-1,3,4-triazoles, and thiazolyl-1,3,4-triazoles (**Th1**–**Th22**), synthesized in our laboratory, according to a previously described procedure [20], were investigated. The structures of the compounds used in this study are presented in Figure 1. All components of the mobile phases used were of analytical grade purity.

### 2.2. Lipophilicity Evaluation of the Studied Compounds

**Figure 2:**
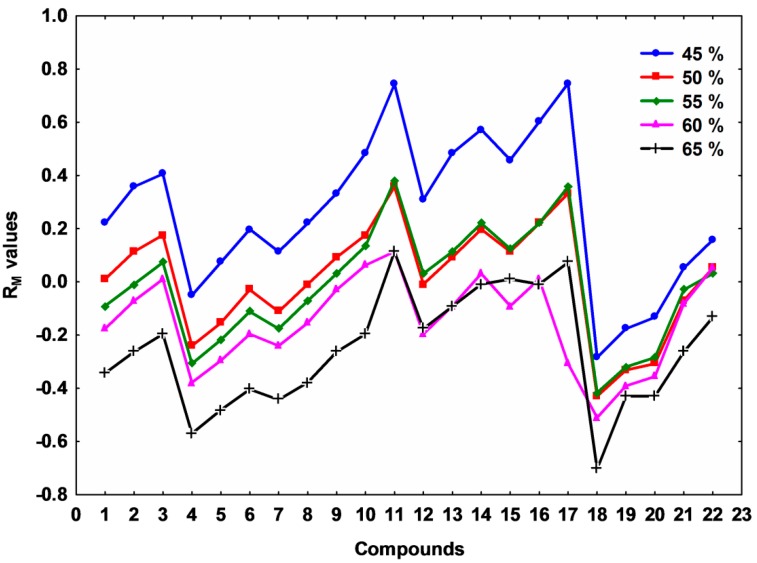
Profiles of R_M_ values at all mobile binary phase composition (% of organic modifier) for all studied compounds.

In all instances, it was found that the retention of the investigated compounds increased with increasing concentration of the organic modifier in the mobile phase. The change of the retardation factor with increasing the organic solvent content in the binary mobile phase, correlates well with the polarity, the substituents (phenyl, allyl, methyl, hydrogen and bromine). The R_M_ values obtained from Equation (1) decrease linearly as the concentration of the organic modifier in the mobile phase increases, and therefore they might be used to assess lipophilicity. The profiles of the R_M_ values present regular changes as the organic modifier content increases, indicating that the same mechanism of lipophilic interaction is dominant (Figure 2). Nevertheless, minor changes of these systematic regularities can be observed with compounds **Th-11-17**, most probably due to their more rigid molecular structures compared to compounds **Th-1-8**.

The results of regression analyses using Equation (2) (intercept values (R_M0_), slopes (b) and correlation coefficient (r) are presented in Table 1. These values afford a quantitative estimation for the distribution of the investigated compounds between a non-polar phase (a chemically bound reversed stationary phase) that represents the biological membranes, and a polar phase that represents the extracellular aqueous environment. As expected, it can be observed that there is a linear dependence between the values of R_M0_ (lipophilic parameter) and the values of b (specific hydrophobic surface) for the majority of these compounds. This linear dependence shows that thiazolyl derivatives might form a homologous series of compounds, as has been suggested by some authors [22]. Once more, it can be observed that compound **Th-1-8** has a mean correlation coefficient of 0.979 ± 0.005, while compounds **Th-9-17 and Th-18-22** have 0.927 ± 0.034 and 0.925 ± 0.018, respectively. In addition, similar significant differences (*p* < 0.0003, 12.56 < *f* < 25.34, ANOVA test) could be observed between these three groups, for R_M0_ and b values (Table 1). Based on these observations and together with structural aspects (Table 1), the studied compounds have been divided in three groups: compounds **Th-1-8** form group A, while compounds **Th-9-17 and Th-18-22** form group B and group C, respectively. This classification will be used in further observations and discussions (*vide infra*).

**Table 1:** Retention parameters obtained from equation R_M_ = R_M0_ + bC.

Group	Compd.	R_M0_	Mean ± SD	b	Mean ± SD	*r* ^a^	Mean ± SD
**A**	**Th-1**	1.369	1.338 ± 0.213	−2.627	−2.623 ± 0.165	**0.987**	0.979 ± 0.005
	**Th-2**	1.595		−2.853		**0.980**	
	**Th-3**	1.600		−2.739		**0.979**	
	**Th-4**	0.986		−2.355		**0.979**	
	**Th-5**	1.161		−2.502		**0.976**	
	**Th-6**	1.398		−2.740		**0.981**	
	**Th-7**	1.195		−2.484		**0.972**	
	**Th-8**	1.398		−2.685		**0.975**	
**B**	**Th-9**	1.479	1.779 ± 0.435	−2.630	−3.012 ± 0.680	**0.965**	0.927 ± 0.034
	**Th-10**	1.754		−2.948		**0.954**	
	**Th-11**	2.020		−3.133		0.912	
	**Th-12**	1.305		−2.463		0.899	
	**Th-13**	1.565		−2.672		0.898	
	**Th-14**	1.630		−2.659		0.876	
	**Th-15**	1.664		−2.905		0.943	
	**Th-16**	1.783		−2.962		0.921	
	**Th-17**	2.808		−4.737		**0.974**	
**C**	**Th-18**	0.540	0.508 ± 0.168	−1.840	−1.354 ± 0.279	**0.951**	0.925 ± 0.018
	**Th-19**	0.286		−1.134		0.920	
	**Th-20**	0.393		−1.283		0.921	
	**Th-21**	0.620		−1.286		0.900	
	**Th-22**	0.701		−1.227		0.931	

^a^ bold = correlation coefficients > 0.95. R_M0_ = lipophilicity estimation parameter; b = slope.

**Figure 3:**
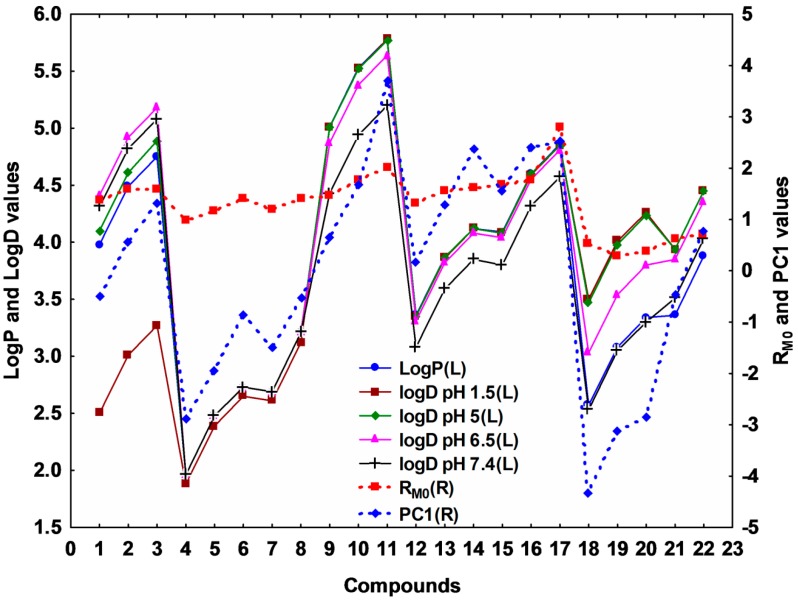
Relationship between computed LogP and LogD (calculated by the equally weighted calculation algorithm combining the three methods described in the Experimental Section) values profiles and experimental lipophilicity indices R_M0_ and PC1. The L or R information in the brackets refers to either left or right disposition of the values on the *y* axis.

Besides the well-known R_M0_ values which are the experimentally-determined indices for the lipophilicity of the studied compounds, PC1 values, which are obtained by applying PCA to the matrix of the R_F_ values for all mobile phases for all compounds, can be successfully used for quantifying the lipophilicity and may bring complementary information to the R_M0_ [14,15]. The variation of these two parameters for the studied compounds, along with the computed LogP and LogD values, is presented in Figure 3. It can be observed that the R_M0_ and PC1 profiles are well correlated with the profiles of the computed LogP and LogD values, all describing similar patterns. Regarding the experimental lipophilicity parameters, R_M0_ and PC1, the compounds present expected variations of the lipophilicity, as the functional groups are modified (both R and X groups).

Analyzing the values obtained for PC1, it was noticed that the cyclisation of thiosemicarbazides **Th-1-8** to the corresponding thiazolyl-triazoles **Th-9-17** increased the lipophilicity, probably as result of a decrease of the polar character of the former, due to the -CO-NH-NH-CS-NH- fragment. Concerning the nature of the Y substituent, it can be observed that the presence of an electron withdrawing substituent (phenyl) increases the lipophilicity, while electron donating groups decrease it (**Th-11** > **Th-14** > **Th-17**) (Figure 3). Regarding the X substituent and the whole molecules, as expected in these homologous series, the lipophilicity increases with the molecular volume, the bromo substituted derivatives being the most lipophilic compounds. Also, the *N-*substituted thiazolyl-triazoles (**Th-9-17**) are more lipophilic then the corresponding thiadiazoles (**Th-21-22**) and oxadiazoles (**Th-18-20**), the molecular volume decreasing in the order: group B > group C (S > O) (Figure 3).

According to their structure (Figure 1), the studied compounds can be involved in acid-base equilibria, and are hence ionizable, so that the analysis of the distribution coefficient (LogD) at several physiological relevant pH levels is adequate. Figure 3 also presents the profiles of the LogD values at four pH levels (stomach (1.5), duodenum (5), jejuno-ileum (6.5) and blood serum (7.4) pH levels). The compounds from group A (**Th-1-8**) have slightly basic behavior; the LogD values increase as the pH increases, with a more pronounced effect, thus a much wider interval of distribution, for the first three compounds—which can be explained by the electron withdrawing inductive effect of the phenyl group. Mainly due to the presence of the thiol group, the compounds from groups B and C (**Th-9-22**) present rather an acidic behavior, which is more evident for compounds **Th-9-11** and in group C (**Th-18-22**). This may suggest that compounds **Th-1-3** are better absorbed in intestine than in stomach, while compounds **Th-9-11** and **Th-18-22** are better absorbed in stomach and less in intestine, due to their higher uncharged population of molecules in the first mentioned compartments. The other compounds (**Th-4-9**, **Th-12-17**) are to a lesser extent affected by pH variation at the studied interval (*i.e.*, 1.5–7.4).

**Figure 4:**
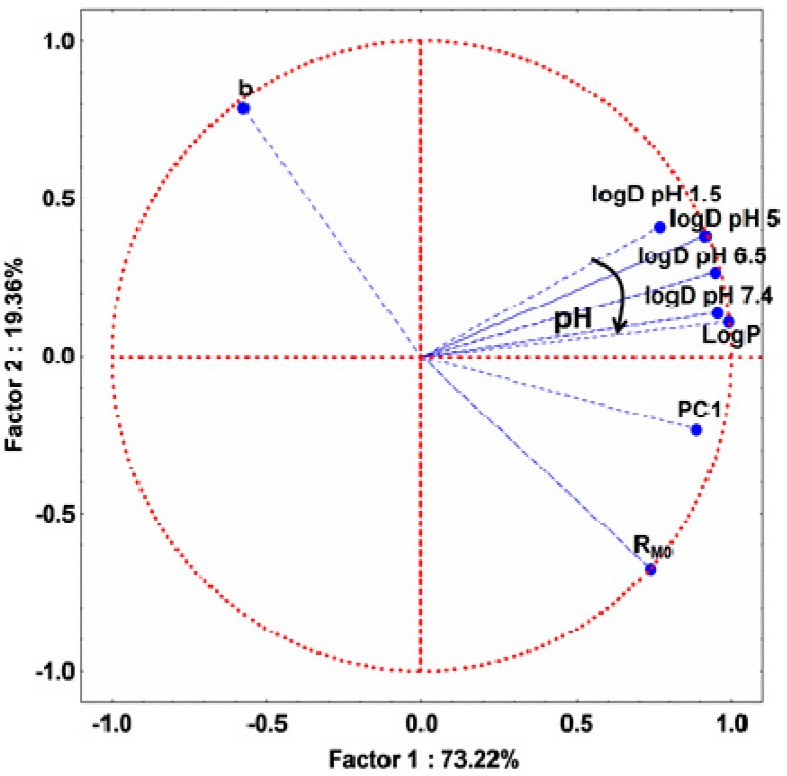
Loadings (circle correlation) for the experimental lipophilicity indices (R_M0_, PC1, b) and computed (logD and logP—calculated by the equally weighted calculation algorithm combining the three methods described in the Experimental Section) lipophilicity indices for all the studied compounds.

**Figure 5:**
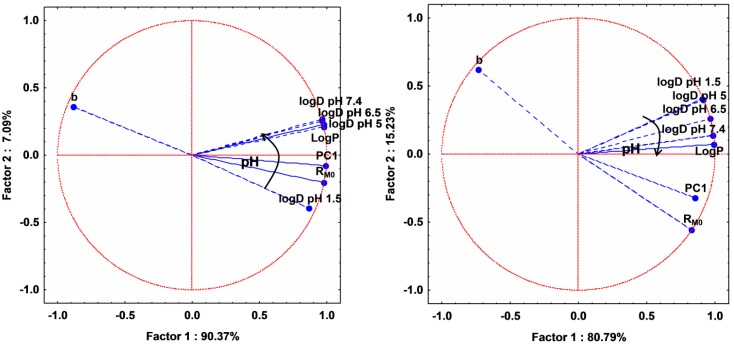
Loadings (circle correlation) for the experimental lipophilicity indices (R_M0_, PC1, b) and computed (LogD and LogP—calculated by the equally weighted calculation algorithm combining the three methods described in the Experimental Section) lipophilicity indices for compounds in group A (**left**) and for compounds in groups B and C (**right**).

Applying PCA on both experimentally and computed lipophilicity indices on all studied compounds, 2D plot of the loadings (circle correlation) showed a regular increase of the correlation between LogD values and experimentally determined lipophilicity indices (PC1, R_M0_) as the pH increases (Figure 4 and Figure S1). However, since the compounds behaved differently according to their group, a PCA application on two separated groups: A (basic compounds) and B and C (acidic compounds) revealed interesting results (Figure 5 and Figure S2). As expected, in both groups, PC1 and R_M0_ positively correlate with each other (*r* = 0.804) and these indices present a strong negative correlation with b (*r* = −0.965 with R_M0_ and *r* = −0.621 with PC1) since it is the characteristic of the specific hydrophobic surface area [6,11,12]. LogP and LogD values afford a relatively moderate correlation with the experimentally determined indices, most probably due to the limitation of estimation of computed indices for complex compounds, since none of the available methods can take into consideration all the effects of molecular conformation. However, as can be observed from Figure 5, LogD at acidic pH in the case of group A and LogD at basic pH in the case of groups B and C are more correlated with R_M0_ and PC1 values.

**Figure 6:**
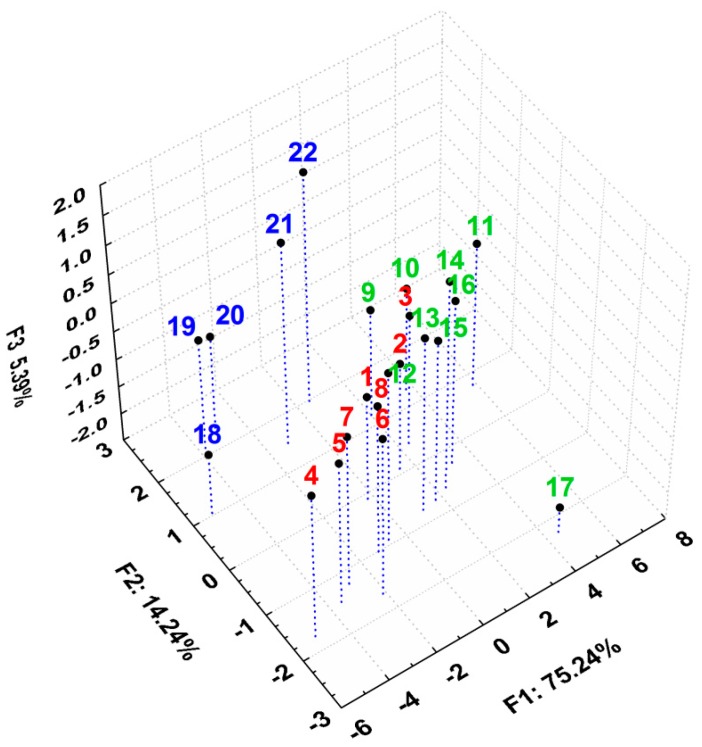
3D plot of the scores for first three principal components after applying PCA on all the discussed parameters, describing lipophilicity for all the studied compounds.

The scatterplot of the scores of the first three principal components after applying PCA on all the previously discussed lipophilicity indices reveals an obvious clustering of the compounds, according to their properties and structural aspects, in very good agreement with the grouping performed in Table 1 (Figure 6). Group A and group B overlap to some extent but group C forms a distinct cluster despite the high scattering within it, most probably due to numerous changes caused by the presence of the heteroatom (Y substituent, Table 1). Compound **Th-17** appeared as an outlier due to its distinct higher R_M0_ value.

### 2.3. Antioxidant and Anti-Inflammatory Effects of the Studied Compounds

The new thiazole compounds have anti-inflammatory effects by lowering the acute-phase response of bone marrow (L and PMN), phagocytic capacity (PI and PA) and oxidative stress [21]. The best anti-inflammatory and antioxidant effects were found for thiazolyl-carbonyl-thiosemicarbazides **Th-1-8** (Group A) and for the oxadiazoles **Th-18-20** and thiadiazole **Th-21** (Group C). Also, the virtual screening of thiazole derivatives revealed that all twenty-two compounds bind the active site of iNOS [21]. Based on the virtual screening and on the obtained results, the activity may be due to their capacity to reduce NO synthesis by blocking the active site of iNOS [21].

In order to obtain a model with less variables, we applied PCA on determined biological parameters (L, PMN, PA, PI, NO, TOS, TAR, OSI) (Table 2).

**Table 2:** Effect of thiazolyl-carbonyl-thiosemicarbazides and thiazolyl-azoles on the determined biological parameters for anti-inflammatory and antioxidant activities.

Comp.	Leukocytes	PMN	PA	PI	NO	TOS	TAR	OSI
**Th-1**	5587.5 ± 455	60.4 ± 4.1	18.5 ± 1.8	21.5 ± 4.6	50.479 ± 5.7	51.622 ± 6.2	1.108 ± 0.003	4.66 ± 2.2
**Th-2**	6543 ± 271	60.6 ± 3.1	22.5 ± 2.8	21.5 ± 2.8	51.827 ± 6.3	63.265 ± 5.4	1.099 ± 0.003	5.76 ± 5.3
**Th-3**	4987 ± 133	63.6 ± 3.9	31 ± 1.8	21.5 ± 0.9	52.838 ± 8.9	39.601 ± 7.9	1.095 ± 0.003	3.62 ± 2.9
**Th-4**	12,218.8 ± 1341	82.8 ± 4.1	29 ± 1.8	22.5 ± 3.5	47.501 ± 2.8	49.823 ± 3.6	1.109 ± 0.006	4.49 ± 3.6
**Th-5**	11,633.3 ± 154	59.8 ± 3.6	22.5 ± 0.9	19.5 ± 2.3	47.024 ± 8.9	51.557 ± 17.1	1.096 ± 0.001	4.7 ± 3.1
**Th-6**	6056.25 ± 862	70.4 ± 2.9	18 ± 1.5	13 ± 3.2	50.591 ± 9.1	43.108 ± 5.6	1.097 ± 0.006	3.93 ± 2.6
**Th-7**	6356.25 ± 66	73.2 ± 4.0	53.5 ± 3.5	20.5 ± 0.9	54.355 ± 6.2	44.073 ± 3.8	1.107 ± 0.003	3.98 ± 3.8
**Th-8**	5718.75 ± 104	66.6 ± 1.0	21 ± 1.1	21.5 ± 0.9	48.793 ± 5.5	55.664 ± 4.2	1.107 ± 0.002	5.03 ± 4.1
**Th-9**	9000 ± 428	67.4 ± 3.0	20.5 ± 0.9	21 ± 7.0	65.479 ± 4.8	68.780 ± 16.2	1.101 ± 0.001	6.25 ± 5.2
**Th-10**	5606.25 ± 154	61.2 ± 3.4	23 ± 1.1	20.5 ± 0.9	65.142 ± 5.3	54.164 ± 5.4	1.095 ± 0.003	4.95 ± 5.4
**Th-11**	11,137.5 ± 483	86.4 ± 2.6	19.75 ± 2.4	21 ± 1.1	66.799 ± 5.9	66.394 ± 8.9	1.092 ± 0.003	6.08 ± 4.9
**Th-12**	8456.25 ± 565	78.6 ± 1.0	20 ± 1.5	22.5 ± 3.5	70.029 ± 6.4	90.592 ± 4.2	1.095 ± 0.004	8.27 ± 4.2
**Th-13**	10,172 ± 331	80.8 ± 3.0	27.5 ± 0.9	25.5 ± 2.8	75.451 ± 6.9	50.886 ± 5.6	1.1 ± 0.012	4.63 ± 3.6
**Th-14**	10,172 ± 520	75.4 ± 4.5	23.5 ± 0.9	28 ± 2.1	86.04 ± 2.9	66.876 ± 9.7	1.097 ± 0.006	6.1 ± 4.7
**Th-15**	9982.5 ± 81	77.4 ± 5.1	29 ± 3.2	24 ± 4.3	71.630 ± 5.9	69.171 ± 4.4	1.094 ± 0.006	6.32 ± 4.4
**Th-16**	9225 ± 333	77.4 ± 1.9	27 ± 1.1	21.2 ± 1.0	63.091 ± 5.1	66.681 ± 9.1	1.103 ± 0.009	6.04 ± 4.1
**Th-17**	10,147.5 ± 528	75.4 ± 5.2	19.5 ± 0.9	15 ± 3.5	60.479 ± 11.8	69.962 ± 5.2	1.093 ± 0.003	6.38 ± 5.2
**Th-18**	4575 ± 805	63.7 ± 2.4	56 ± 8.6	17 ± 1.9	43.23 ± 3.9	78.089 ± 14.1	1.118 ± 0.006	6.98 ± 4.1
**Th-19**	7443.75 ± 1172	72.8 ± 5.0	13.5 ± 2.78	20 ± 1.5	45.198 ± 2.2	79.849 ± 12	1.119 ± 0.009	7.13 ± 5.9
**Th-20**	4837.5 ± 1305	59.2 ± 5.6	20 ± 1.5	16 ± 3.0	43.288 ± 5.0	81.022 ± 13.2	1.106 ± 0.003	7.32 ± 3.2
**Th-21**	3000 ± 241	52.6 ± 2.1	23 ± 1.9	14.5 ± 4.6	43.737 ± 6.2	67.137 ± 10.6	1.114 ± 0.007	6.03 ± 4.5
**Th-22**	5812.5 ± 399	66.4 ± 0.8	23.5 ± 0.9	35 ± 8.2	52.08 ± 8.1	56.694 ± 7.9	1.105 ± 0.001	5.13 ± 4.9

**Figure 7:**
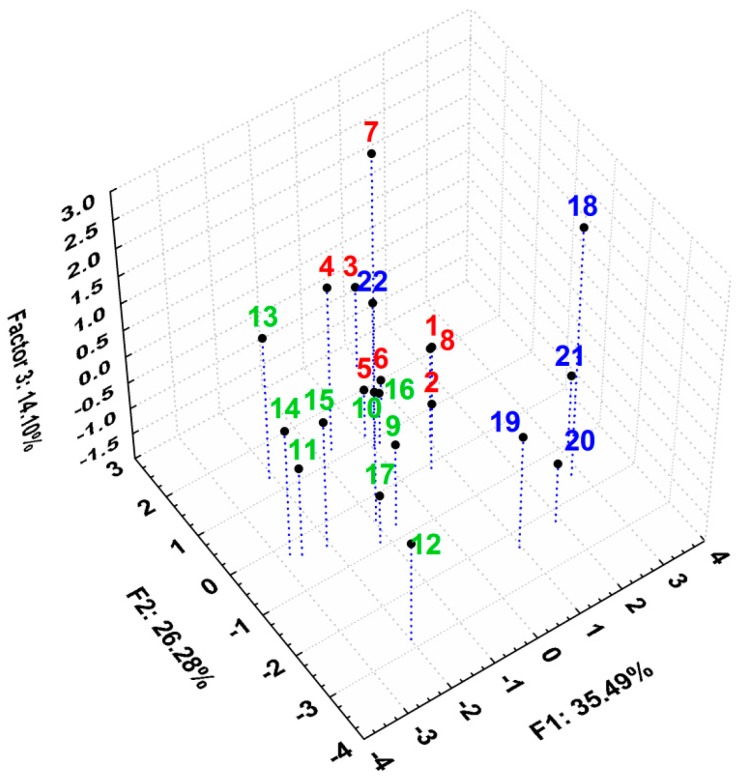
3D scatterplot of the scores for the first three principal components after applying PCA on all the discussed parameters, describing biological activity for all the studied compounds.

After applying PCA only on these biological activity parameters, an acceptable model was obtained, the first three principal components containing 75.87% of total variance (Figure S3). However, it is interesting that the 3D scatterplot of the first three principal components revealed a pattern of clustering of the studied compounds, similar to the one of their lipophilicity (Figure 7). The three groups are well and distinctly grouped, with compound **Th-22** as an exception. Here, groups A and B are better separated than in case of the lipophilicity (Figure 6), indicating their more distinct behavior in case of their biological activities.

### 2.4. Comparative Evaluation of Both Lipophilicity Indices and Antioxidant and Anti-Inflammatory Activities Evaluation for the Studied Compounds

**Figure 8:**
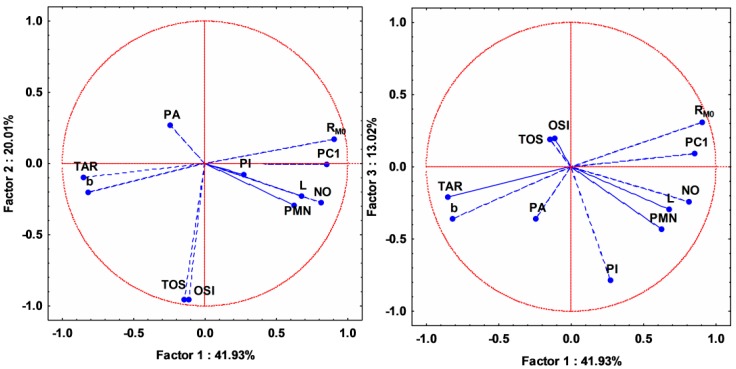
Loadings (circle correlation) for the assessed biological activity (**left panel**) and lipophilicity indices (**right panel**) for all studied compounds. L = leukocytes; PMN = polymorphonuclear leukocytes; PA = phagocytic activity; PI = phagocytosis index; NO = nitric oxide; TOS = total oxidative status; TAR = total antioxidant response; OSI = oxidative stress index; R_M0_ = lipophilicity estimation parameter; b = slope; PC1 = first principal component.

A similar clustering of the compounds to the one seen in the case of their lipophilicity indicates that there are some possible correlations between their lipophilicity and their biological activity. In this regard, PCA was applied on all the studied variables, describing lipophilicity and the anti-inflammatory and antioxidant activities. After applying PCA, an acceptable model was obtained—the first three principal components containing 74.96% of total variance (Figure S4). Analyzing the loadings of the variables and the matrix correlation of the original variables, we observed that NO parameter is positively correlated with the lipophilicity, and TAR activities are negatively correlated with the lipophilicity indices (Figure 8, Table 3).

**Table 3:** Correlation matrix of the lipophilicity indices and antioxidant and anti-inflammatory effects for all the studied compounds. Values greater than 0.5 are in bold face characters.

	L	PMN	PA	PI	NO	TOS	TAR	OSI	R_M0_	b	PC1
L	1.000	**0.735**	−0.175	0.250	**0.522**	0.025	−0.455	0.043	0.453	−0.469	0.313
PMN		1.000	0.016	0.281	**0.576**	0.086	−0.305	0.098	0.398	−0.384	0.354
PA			1.000	−0.008	−0.111	−0.162	0.291	−0.174	−0.166	0.071	−0.318
PI				1.000	0.443	−0.105	−0.127	−0.098	−0.009	0.153	0.320
NO					1.000	0.109	**−0.629**	0.135	**0.600**	−0.474	**0.741**
TOS						1.000	0.178	**0.999**	−0.206	0.208	−0.091
TAR							1.000	0.142	**−0.782**	**0.704**	**−0.741**
OSI								1.000	−0.178	0.184	−0.063
R_M0_									1.000	**−0.965**	**0.804**
b										1.000	**−0.621**
PC1											1.000

L = leukocytes; PMN = polymorphonuclear leukocytes; PA = phagocytic activity; PI = phagocytosis index; NO = nitric oxide; TOS = total oxidative status; TAR = total antioxidant response; OSI = oxidative stress index; R_M0_ = lipophilicity estimation parameter; b = slope; PC1 = first principal component.

**Figure 9:**
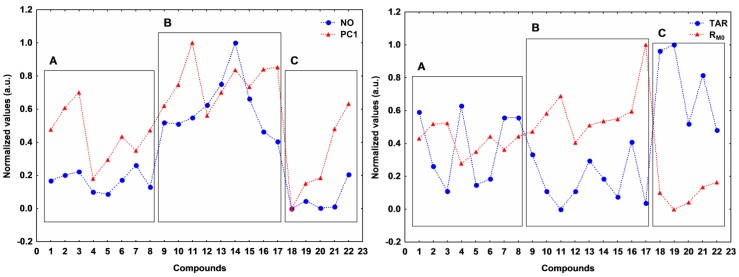
Profiles of NO *vs.* PC1 (**left panel**) and TAR *vs.* R_M0_ (**right panel**) for all studied compounds.

Besides this, PA, PI, TOS and OSI have statistically no correlation, while PMN and L have low correlation with any lipophilicity indices. However, the two previously mentioned variables (TAR and NO) have distinctly higher correlation with all lipophilicity indices. Activation and stimulation of phagocytes in the inflammatory process is dependent on membrane-derived lipid mediators (e.g., arachidonic acid derivatives) [23,24]. This may explain the positive correlation between the reactive nitrogen intermediates (RNIs) (*i.e.*, NO), produced by phagocytes, and lipophilicity (Figure 8 and Figure 9). On the other hand, as a consequence of oxidative stress increase by reactive oxygen intermediates (ROIs) and RNIs, the antioxidant defense capacity is reduced. This justifies the negative correlation between TAR and lipophilicity (Figure 8 and Figure 9).

**Figure 10:**
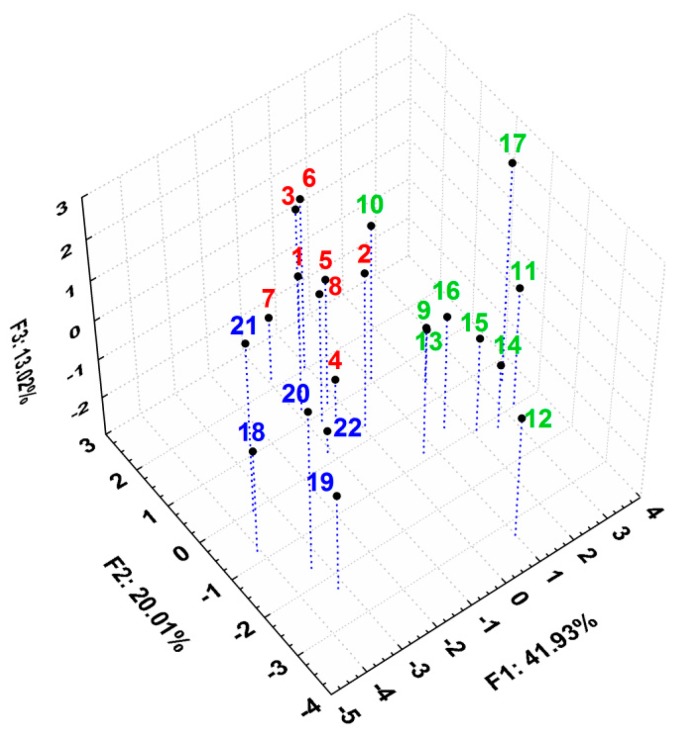
3D scatterplot of the scores for the first three principal components after applying PCA on all the discussed parameters describing biological activity and lipophilicity for all studied compounds.

The 3D scatterplot of the scores for the first three principal components reported in Figure 10 reveals an even better and more compact clustering for the studied compounds forming the three distinct groups: the thiosemicarbazides **Th-1-8** from group A with the best anti-inflammatory and antioxidant effects [21], the triazoles **Th-9-17** from group B and the oxadiazoles and thiadiazoles **Th-18-22** from group C.

The knowledge of the grouping patterns of the tested variables allows for the reduction of experimental determined parameters, the variables which are close to each other in PCA results describing similar properties of the substances. For this reason, it is not necessary to measure and evaluate all variables to achieve the same characterization—thus reducing the amount of experimental work required [25].

The pattern of the profiles of both NO and TAR *vs.* PC1 and R_M0_ respectively, clearly sustain the positive and negative correlation (Figure 9). For the first case (NO *vs.* PC1), the A and C groups present a distinctly higher similarity of the pattern than the B group (Figure 9 left), and the NO synthesis increases with the lipophilicity in agreement with the previous observations. Thus, best anti-inflammatory compounds **Th-1-8** have the medium values for the lipophilicity parameters. For the second case, the negative correlation profiles between TAR and R_M0_ (Figure 9 right) is salient for group A and for some compounds from groups B and C. The negative correlation might be explained by the fact that the increase in hydrophobicity of a given compound may imply an increase in redox potential, thus decreasing its antioxidant ability [26].

## 3. Experimental Section

### 3.1. Chromatographic Procedure

Chromatography was performed on 20 × 20 cm RP-18F_254s_ TLC precoated silica plates (Merck; Darmstadt, Germany). Solutions (1 mg/mL) of the tested compounds were prepared in *iso*-propanol, and 3 μL aliquots were spotted in duplicate on the plates by hand, 10 mm from the bottom edge and 20 mm apart. The mobile phases were composed of the *iso*-propanol-water binary mixtures, with a varying content of organic modifier between 45% and 65% (*v*/*v*) in 5% increments, as the study compounds differed considerably in their retention. Chromatography was performed in a normal developing chamber at room temperature, the developing distance being 10 cm. The chromatography chamber was saturated with the mobile phase for 30 min before use. After the development (30–60 min), the plates were air dried at room temperature and examined under a UV lamp (λ = 254 nm), and the R_F_ (retardation factor) values were measured manually by a digital caliper. The experiments were performed in triplicate.

### 3.2. Prediction of Partitioning and Distribution Coefficients

The lipophilicity effects of compounds were predicted as log P and log D using the partitioning module of MarvinSketch 5.5.0.1, 2011 (ChemAxon Kft., Budapest, Hungary, http://www.chemaxon.com). Calculations for log D were estimated for the implicit reference values: log D_1.5_ (log D calculated at the physiological pH of stomach), log D_5.0_ (log D calculated at the physiological pH of duodenum), log D_6.5_ (log D calculated at the physiological pH of jejuno-ileum) and log D_7.4_ (log D calculated at the physiological pH of blood serum). Both calculations for log P and log D were performed using four different methods: a calculation algorithm developed by ChemAxon Kft. based on the publication of Viswanadhan and coworkers [27], one based on method developed by Klopman and coworkers [28], another one using the log P data from PHYSPROP© database and an equally weighted calculation algorithm combining the three methods. Supplementary, the software was set to take in account, for each set of calculations/algorithm, the major tautomeric forms of each compound.

### 3.3. Biological Activity Measurements

The synthesized thiazolyl-carbonyl-thiosemicarbazides and thiazolyl-azoles **Th-1-Th-22** were previously evaluated *in vivo* for their anti-inflammatory activity in a turpentine oil-induced inflammation model [21]. Their anti-inflammatory activity was assessed by evaluating the acute-phase response of bone marrow by leukocytes count (L) and by differential leucocyte count, expressed as a percentage (PMN) and by phagocytes’ capacity (PI%-phagocytosis index, PA-phagocytic activity), nitric oxide (NO) synthesis and antioxidant capacity (TOS-total oxidative status, TAR-total antioxidant response and OSI-oxidative stress index [21].

### 3.4. Statistical Analysis

All results were expressed as mean ± standard deviation (SD) of three independent experiments. Statistical comparisons between the groups were performed using one-way analysis of variance (ANOVA) test. A value of *p* < 0.05 was considered to be statistically significant.

## 4. Conclusions

The applied PCA method of multivariate analysis allowed us to compare the chromatographic retention data, the lipophilic parameters and the biological parameters of the investigated thiazolyl-carbonyl-thiosemicarbazides and thiazolyl-azoles.

The obtained results illustrate how the nature of the substituents attached to a pharmacophore (thiazole for group A, thiazolyl-azole for groups B and C) influences the chromatographic retention, *i.e.*, the lipophilic behavior and the determined biological parameters of the investigated compounds.

The PCA method could be used to group the studied compounds based on the influence of the substituents on the lipophilic character of the whole molecule. Also, the results of the PCA analysis applied on all the experimental and computed lipophilic parameters and on the biological parameters show, as expected, that the best anti-inflammatory and antioxidant compounds are correlated with medium values for the lipophilicity parameters (an optimum hydrophilic-lipophilic balance). The knowledge of the grouping patterns of the tested variables offers the possibility to reduce the number of determined parameters, needed for predicting biological activity.

## Supplementary Materials

Supplementary materials can be accessed at: http://www.mdpi.com/1420-3049/20/12/19841/s1.
