# Supplementary material for: Study of the Relationships between the Structure, Lipophilicity and Biological Activity of Some Thiazolyl-carbonyl-thiosemicarbazides and Thiazolyl-azoles
Source: Molecules. 2015 Dec 11;20(12):22188–201. doi: 10.3390/molecules201219841 (PMC6332165; doi:10.3390/molecules201219841)
Supplement: Supplementary file 1 [file molecules-20-19841-s001.pdf]

Supplementary Material: A Comparative Study between the Lipophylicity of Some Thiazolyl-Carbonyl-Thiosemicarbazides and Thiazolyl-Azoles and Their Anti-Inflammatory and Antioxidant Activities

Radu Tamaian, Augustin Moţ, Radu Silaghi-Dumitrescu, Ioana Ionuţ, Anca Stana, Ovidiu Oniga, Cristina Nastasă, Daniela Benedec and Brînduşa Tiperciuc

Contents of Supporting Information

Figure S1. Relationships between experimental lipophilicity indices profiles and LogP profile for the studied compounds.
Figure S2. Biplot of the first two principal components using only the lipophilicity parameters.
Figure S3. Biplot of first two principal components using only the biological activity values.
Figure S4. Biplot of first two principal components using both the biological activity and lipophilicity values.

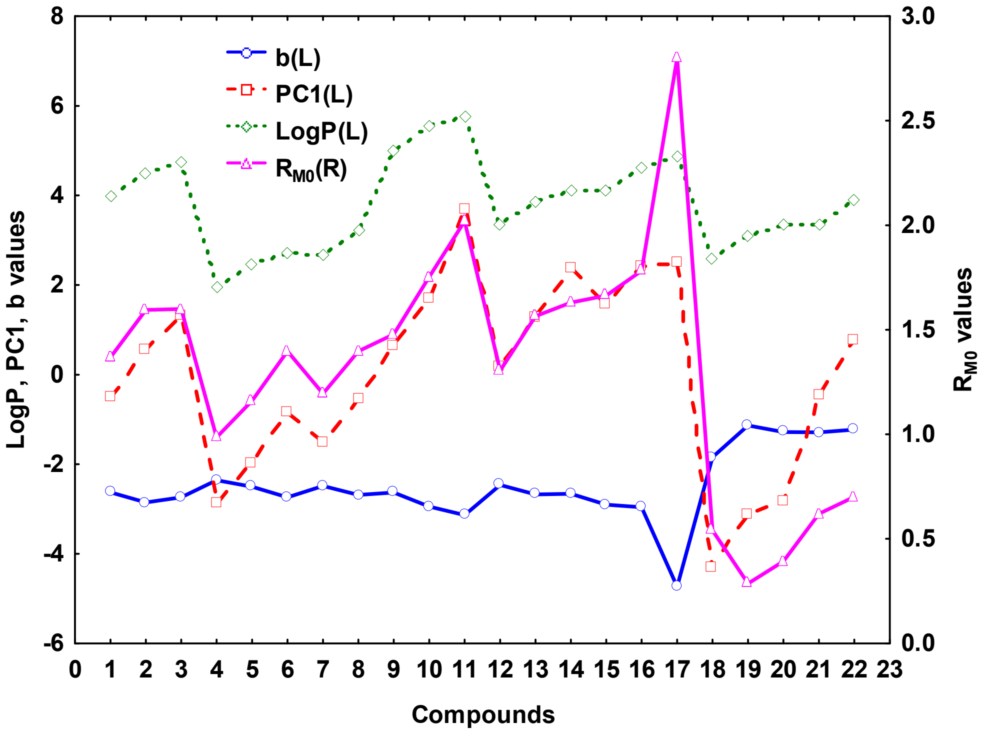

Figure S1. Relationship between experimental lipophilicity indices profiles and log P profile for the studied compounds.

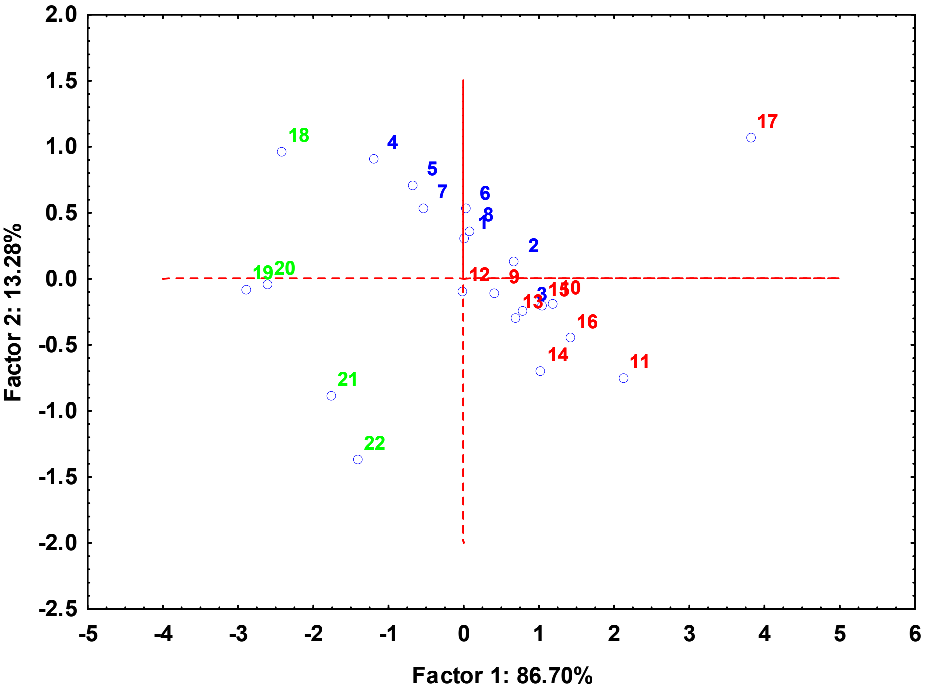

Figure S2. Biplot of first two principal components using only the lipophicity parameters.

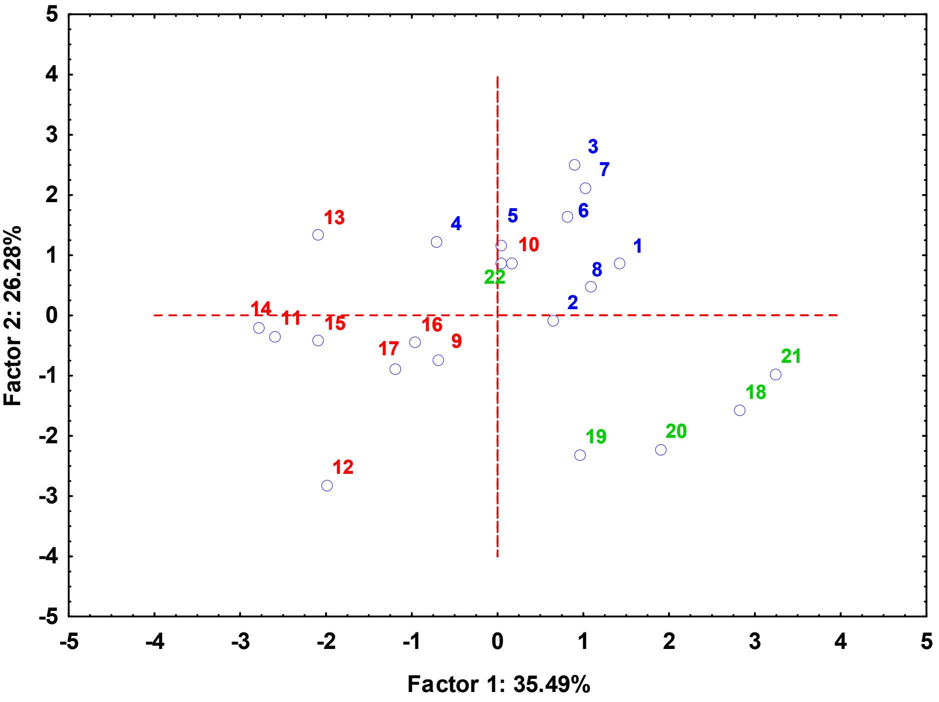

Figure S3. Biplot of first two principal components using only the biological activity values.

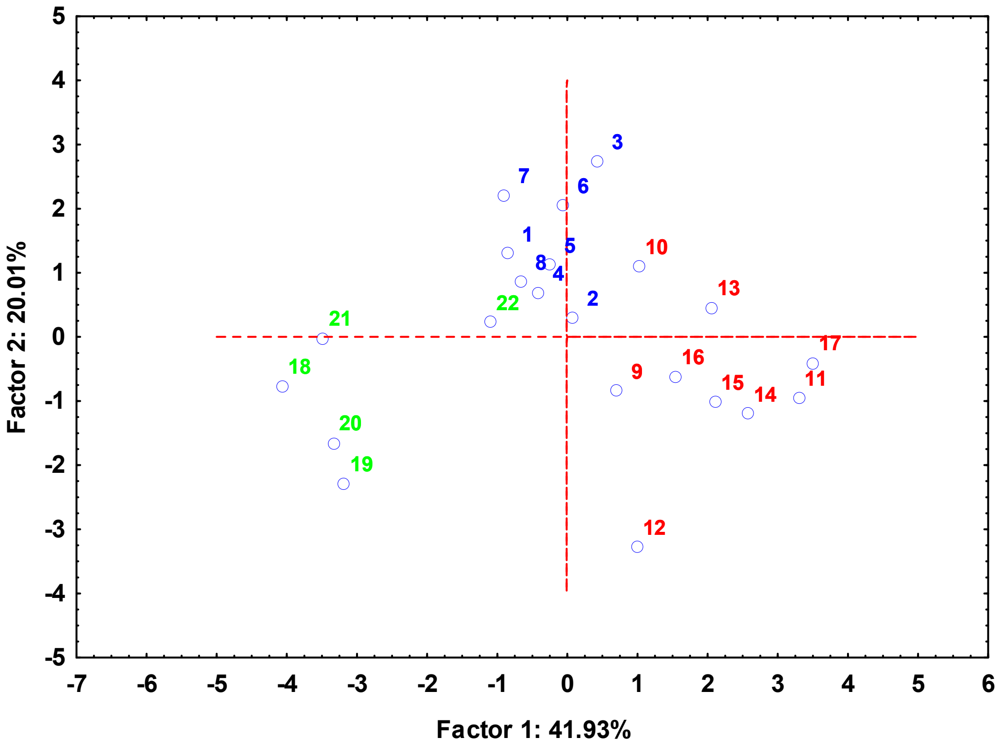

Figure S4. Biplot of first two principal components using both the biological activity and lipophilicity values.
